# Anti-CD74 IgA as a potential biomarker in axial spondyloarthritis: diagnostic utility and clinical implications

**DOI:** 10.1007/s10067-025-07695-3

**Published:** 2025-12-04

**Authors:** Gehad Elsayed Mohammed Ammar, Ali Eed Eldeeb, Heba Ahmed Mourad, Samar AbdAlhamed Tabra

**Affiliations:** 1 Department of Rheumatology and Rehabilitation, Faculty of Medicine, Tanta University, El-Geish Street, Tanta, Gharbia Egypt; 2 Department of Clinical Pathology, Faculty of Medicine, Tanta University, Tanta, Egypt

**Keywords:** Anti-CD74 antibody, Axial spondyloarthritis, Disease activity

## Abstract

**Background:**

Axial spondyloarthritis (axSpA) is chronic autoimmune disease affecting axial skeleton. Recently autoantibodies against CD74 have been linked to the pathogenesis of axSpA.

**Objectives:**

Investigate the diagnostic value of anti-CD74 in Egyptian axSpA patients and their relation to disease activity.

**Methods:**

This study included 30 axSpA patients and control groups, which included 30 patients with peripheral psoriatic arthritis, and 30 healthy volunteers. disease activity was assessed using ASDAS-CRP (Ankylosing Spondylitis Disease Activity Score) and BASDAI (bath ankylosing spondylitis disease activity index) for axSpA patients. Bath ankylosing spondylitis functional index (BASFI) was used for functional assessment and mobility was assessed using Bath Ankylosing Spondylitis Metrology Index (BASMI). ESR, CRP and serum anti-CD74 IgA were evaluated.

**Results:**

There was no significant difference between the three studied groups regarding sex, age, and BMI. The serum levels of anti-CD74 IgA antibodies were significantly higher in axSpA patients compared to control groups.73.3, 66.6 and 3.3% of the axSpA, Peripheral psoriatic arthritis and healthy group, respectively, were anti- CD74 IgA antibody positive. Its specificity was 96.67% compared to the healthy control group, and 33.3% specificity compared to the peripheral psoriatic arthritis group. There was no significant correlation between anti-CD74 IgA antibodies and age, sex, duration of the disease, acute phase reactant, ASDAS-CRP, BASDAI, BASFI, and BASMI.

**Conclusions:**

IgA anti-CD74 may be a useful marker for identifying axSpA. Further research on patients with peripheral SpA is required to determine the practical benefits of this biomarker.

**Table:** 

**Key Points** • *Patients with axSpA had a significantly greater serum level of IgA anti-CD74 than controls*. • *IgA Anti-CD74 had excellent discriminatory power in distinguishing axSpA from healthy controls but fair discriminatory power in distinguishing axSpA from peripheral psoriatic arthritis*. • *IgA anti-CD74 wasn’t correlated with acute phase reactant or disease activity*.	

## Introduction

Axial spondyloarthritis (axSpA) is a chronic inflammatory rheumatic disease which affect axial skeleton mainly [1].

AxSpA is divided into radiographic axSpA and non-radiographic axSpA according to the presence or absence of structural changes in the sacroiliac joints in x ray [2].

The diagnosis of AxSpA in early cases is challenging due to the non-specific nature of its main symptom and lack of specific tests [3, 4].

The inclusion of magnetic resonance images in the new Assessment in SpondyloArthritis International Society (ASAS) criteria leads to early detection of axSpA patients [2, 5].

Although the sensitivity and specificity of HLA-B27 are good, up to 10% of healthy people have positive results. Also, its prevalence differs among ethnic groups and geographical areas [6].

Therefore, new biomarkers are required for early diagnosis and evaluation of the response to treatment and prognosis of the disease. Autoantibodies against CD74 have been linked in recent research to the pathogenesis of axSpA [7].

CD74 is also known as the gamma chain or invariant chain of human histocompatibility leucocyte antigen (MHC) class II. CD74 comprises an N-terminal cytoplasmic region and a C-terminal extracellular Domain. It has two extracellular Domains, thyroglobulin type 1 and class II-associated invariant chain peptide (CLIP), which prevent premature binding of peptide loading by blocking the peptide binding groove in newly assembled class II HLA molecules [8].

CD74 acts as a receptor for macrophage migration inhibitory factor (MIF). The binding of CD74 to macrophage migration inhibitory factor causes activation of nuclear factor kappa B, which is involved in inflammation and cell survival [9]. MIF has been shown to be upregulated in the sera of patients with axSpA [10] and to stimulate osteoblasts in vitro [11].

However, knowledge about the diagnostic value of anti-CD74 is still not confirmed. This study aims to investigate the diagnostic value of anti-CD74 in Egyptian axSpA patients and their relation to disease activity.

## Methods

It's a case–control study conducted at a single center.

### Setting

Patients were recruited from the outpatient clinic of the Rheumatology and Rehabilitation Department, Tanta University Hospitals.

### Patients

The study included thirty adult patients with axSpA who fulfilled ASAS classification criteria [2] and control groups, which included 30 adult patients with peripheral psoriatic arthritis who fulfilled CASPAR [12] criteria for PsA, and 30 healthy volunteers matched for age and gender. Patients who had other rheumatological disorders, were less than 18 years old, had spinal surgery, or had a spinal tumor were excluded from the study.

Duration of the study: twelve months. (From october 2023 to october 2024). Sample size: based on previous research [13], 21 in each group were required to achieve 80% study power at 95% confidence level with a margin of error equal to 5%. The sample size was increased to 30 cases in each group.

### Ethics approval and consent to participate

The institution's ethics board has approved this study with permission number 36264MS333/9/23 and is following the Declaration of Helsinki's ethical principles as well as the ethical standards of the Tanta Faculty of Medicine. Informed consent was received from each patient. Every patient file had a code number that incorporated the results of all investigations, ensuring the privacy of all patient data.

### Clinical assessment

Demographic data and a thorough medication history were recorded. Disease Activity in axSpA patients was assessed using ASDAS-CRP [14] and BASDAI [15]. Level of activity in ASDAS-CRP was divided into inactive disease (< 1.3), low disease activity (1.3- 2.1), high disease activity (2.1–3.5), very high disease activity (˃3.5). BASDAI ≥ 4 was considered an active disease. Functional assessment was done using Bath ankylosing spondylitis functional index (BASFI) [16] mobility was assessed using Bath Ankylosing Spondylitis Metrology Index (BASMI) [17].

### Laboratory assessment

- Routine laboratory assessment: erythrocyte sedimentation rate (ESR) by Westergren method and CRP.
- HLAB27
- Anti-CD74 IgA: blood samples were collected and then the serum was separated by centrifugation. The sample was stored at –20 °C until testing. The Human anti-CD74 IgA level was measured using double antigen sandwich enzyme linked immunosorbent assay (ELISA). The process began by adding the sample to wells precoated with human anti-CD74 IgA antigen, followed by incubation to allow binding. Biotin labeled anti-CD74 IgA antigen was then added, forming a complex with the captured antibodies, and streptavidin-HRP was added to bind to biotin. After incubation and washing to remove unbound components. Chromogen solutions A and B were added, turning the solution blue. An acid was then used to stop the reaction, changing the color to yellow. The intensity of the yellow color was directly proportional to the concentration of Human anti-CD74 IgA in the sample, enabling quantitative measurement.

The collected data were subsequently subjected to statistical analysis as outlined below.

### Statistical analysis

Data was statistically analyzed using SPSS version 20 [18]*.* Qualitative data were described using numbers and percentages. The Kolmogorov–Smirnov& Shapiro–Wilk test was used to verify the normality of distribution. Quantitative data were described using mean, standard deviation, median and interquartile range (IQR). The chi-square test was used for comparing categorical data. Fisher’s Exact or Monte Carlo correction was used in the Correction for chi-square when more than 20% of the cells have an expected count less than 5. The ANOVA test was used for normally distributed quantitative variables to compare more than two groups, and post hoc test for pairwise comparisons. On the other hand, the Kruskal–Wallis test was used to compare more than two groups for not normally distributed quantitative variables. Correlation between variables was done by Spearman's correlation coefficient. P *p*-value less than 0.05 was considered statistically significant.

## Results

This study included 30 axSpA patients and control groups, which included 30 adult patients with peripheral psoriatic arthritis. There was no significant difference between axSpA patients and controls as regard age and sex. In axSpA patients 36.7% had uveitis, 6.7 had inflammatory bowel disease and 46.7 had enthesopathy. 20 patients (66.7%) had radiographic axSpA and 10 patients (33.6%) had non-radiographic axSpA. Nine patients had low disease activity, 9 had high disease activity, while 12 patients had very high disease activity. The demographic and disease-related characteristics of the patients and controls were mentioned in Table 1.

**Table 1:** Demographic and disease-related characteristics of the patients and controls

	axSpA patients (*n* = 30)		PsA patients (*n* = 30)		Controls (*n* = 30)		Test of Sig	p
	No	%	No	%	No	%		
Sex								
Male	19	63.3	12	40.0	13	43.3	χ^2^ = 3.824	0.148
Female	11	36.7	18	60.0	17	56.7		
Age (years)								
Min. – Max	21.0–58.0		24.0–62.0		19.0–49.0		F = 1.1869	0.160
Mean ± SD	37.63 ± 9.82		39.0 ± 11.34		34.10 ± 9.10			
Marital state								
Single	6	20.0	3	10.0	7	23.3	χ^2^ = 3.252	^MC^p = 0.540
Married	24	80.0	26	86.7	22	73.3		
Widow	0	0.0	1	3.3	1	3.3		
Smoking								
No	2	6.7	13	43.3	22	73.3	χ^2^ = 33.673*	< 0.001*
Active	19	63.3	7	23.3	8	26.7		
Passive	9	30.0	10	33.3	0	0.0		
family history of rheumatic diseases	11	36.7	2	6.7	3	10.0	χ^2^ = 11.098*	0.004*
History of					NA	NA		
Uveitis	11	36						
IBD	2	6.7						
enthesitis	14	46.7						
Disease activity	ASDAS-CPR • Inactive:0 • Low disease activity:9 • High disease activity:9 • Very high disease activity:12 BASDAI • Active:14 • Inactive:16		DAPSA • Inactive:4 • Low disease activity:8 • moderate disease activity:11 • high disease activity:7					
BASMI	3.76 ± 1.87		-		-			
Functional assessment (BASFI)	5.42 ± 2.16		-		-			

*axSpA* axial spondyloarthritis, *PsA* psoriatic arthritis, *ASDAS*: Ankylosing Spondylitis Disease Activity Score, BASDAI bath ankylosing spondylitis disease activity index, *DAPSA* Disease Activity Index for Psoriatic Arthritis, *BASFI*: Bath ankylosing spondylitis functional index. *IQR:* Interquartile range

*Significant *p* value if < 0.05

The serum levels of anti-CD74 IgA antibodies were significantly higher in axSpA patients compared to control groups (Peripheral psoriatic arthritis patients and healthy people). 73.3, 66.6 and 3.3% of the axSpA, Peripheral psoriatic arthritis and healthy group, respectively, were anti- CD74 IgA antibody positive based on the cut-off point of anti-CD74 IgA antibodies (> 74 ng/ml) (Table 2).

**Table 2:** Comparison between the three studied groups according to ANTI-CD74

	Group I (*n* = 30)		Group II (*n* = 30)		Group III (*n* = 30)		Test of Sig	*p*
	No	%	No	%	No	%		
IgG4 Anti-CD74								
Negative	8	26.7	10	33.3	29	96.7	χ^2^ 30.951*	< 0.001*
Positive	22	73.3	20	66.6	1	3.3		
Min. – Max	30.00–905.0		21.20–310.0		10.30–88.50		H = 57.687*	< 0.001*
Mean ± SD	232.5 ± 198.6		87.32 ± 74.39		21.93 ± 15.10			
Median (IQR)	159.5 (73.0–371.0)		59.90 (38.0–102.0)		16.45 (13.60–26.60)			
Sig. bet. grps	p_1_ = 0.006*, p_2_ < 0.001*, p_3_ < 0.001*							

*IQR* Inter quartile range, *SD* Standard deviation χ^2^ Chi square test

H: H for Kruskal Wallis test, Pairwise comparison bet. each 2 groups was done using Post Hoc Test (Dunn's for multiple comparisons test)

*p*: *p* value for comparing between the three studied groups

*p*_1_: *p* value for comparing between Group I and Group II

*p*_2_: *p* value for comparing between Group I and Group III

*p*_3_: *p* value for comparing between Group II and Group III

^*^Significant *p* value if < 0.05

Group I: Axial SpA patients

Group II: Peripheral psoriatic arthritis

Group III: Healthy controls

When considering the axSpA HLA-B27 negative subpopulation (Table 3), IgA anti-CD74 were positive in 9 additional/14 axSpA patients with no significant difference between HLA-B27 positive and negative patients regarding the presence of IgA anti-CD74.

**Table 3:** IgA anti-CD74 results according to HLA-B27 status in axSpA patients

	HLA B27					
	Negative (*n* = 14)		Positive (*n* = 16)		χ^2^	^FE^p
	No	%	No	%		
IgA Anti-CD74						
Negative	5	35.7	3	18.8	1.099	0.417
Positive	9	64.3	13	81.3		

χ^2^ Chi square test, *FE* Fisher Exact test

Significant *p* value if < 0.05

Twenty patients have radiographic axSpA and 10 patients have non-radiographic axSpA when considering the presence of IgA anti-CD74, 16 patients (80%) out of twenty patients with radiographic axSpA have positive IgA anti-CD74 and 6 (60%) out of 10 patients with non-radiographic axSpA have positive IgA anti-CD74 with no statistically significant difference between radiographic and non-radiographic axSpA regarding the presence of IgA anti-CD74 (*P* = 0.384) (data not shown).

**Fig. 1:**
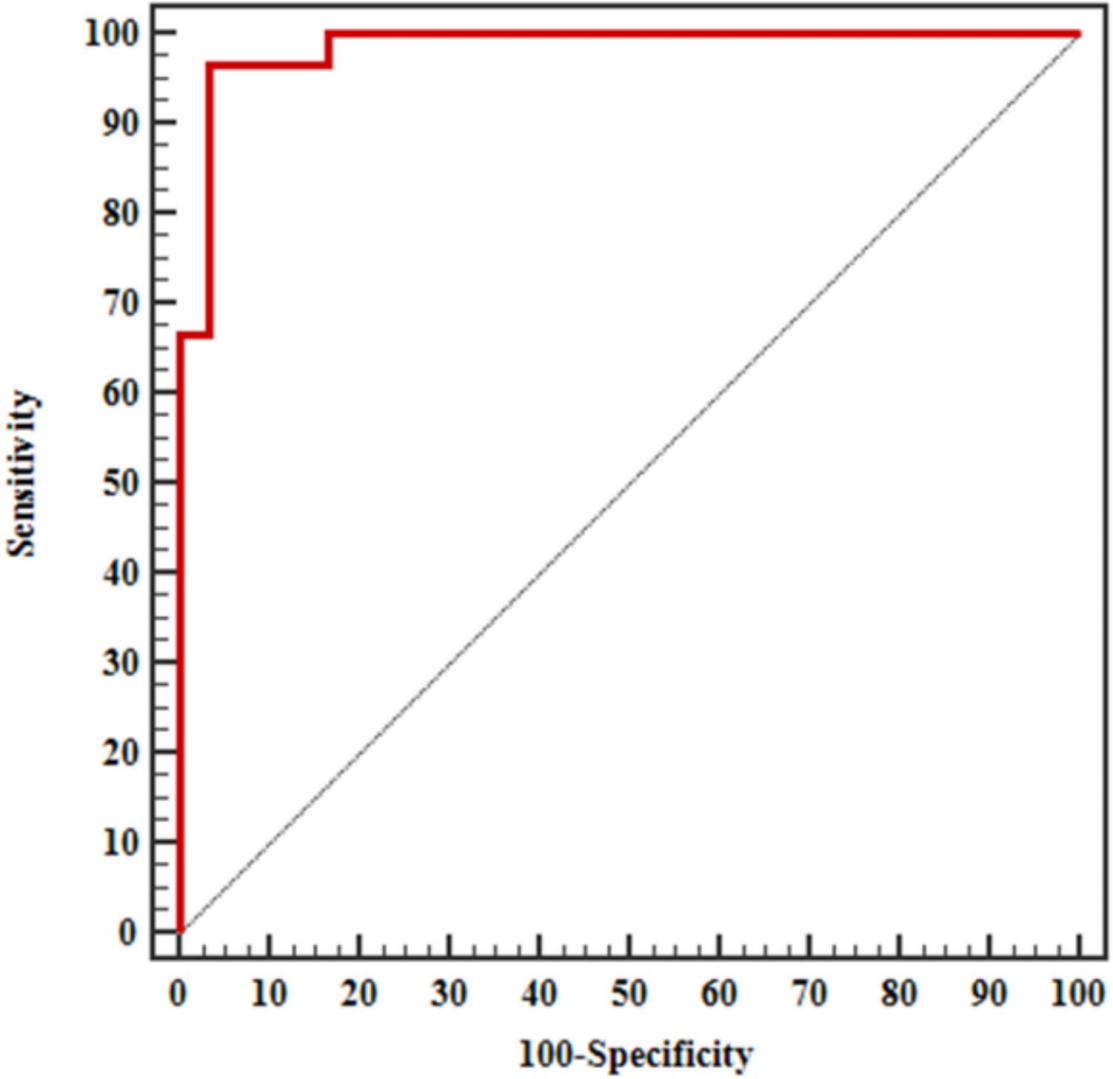
The ROC curve shows that IgA Anti-CD74 has excellent discriminatory power, in distinguishing axSpA from healthy controls

**Fig. 2:**
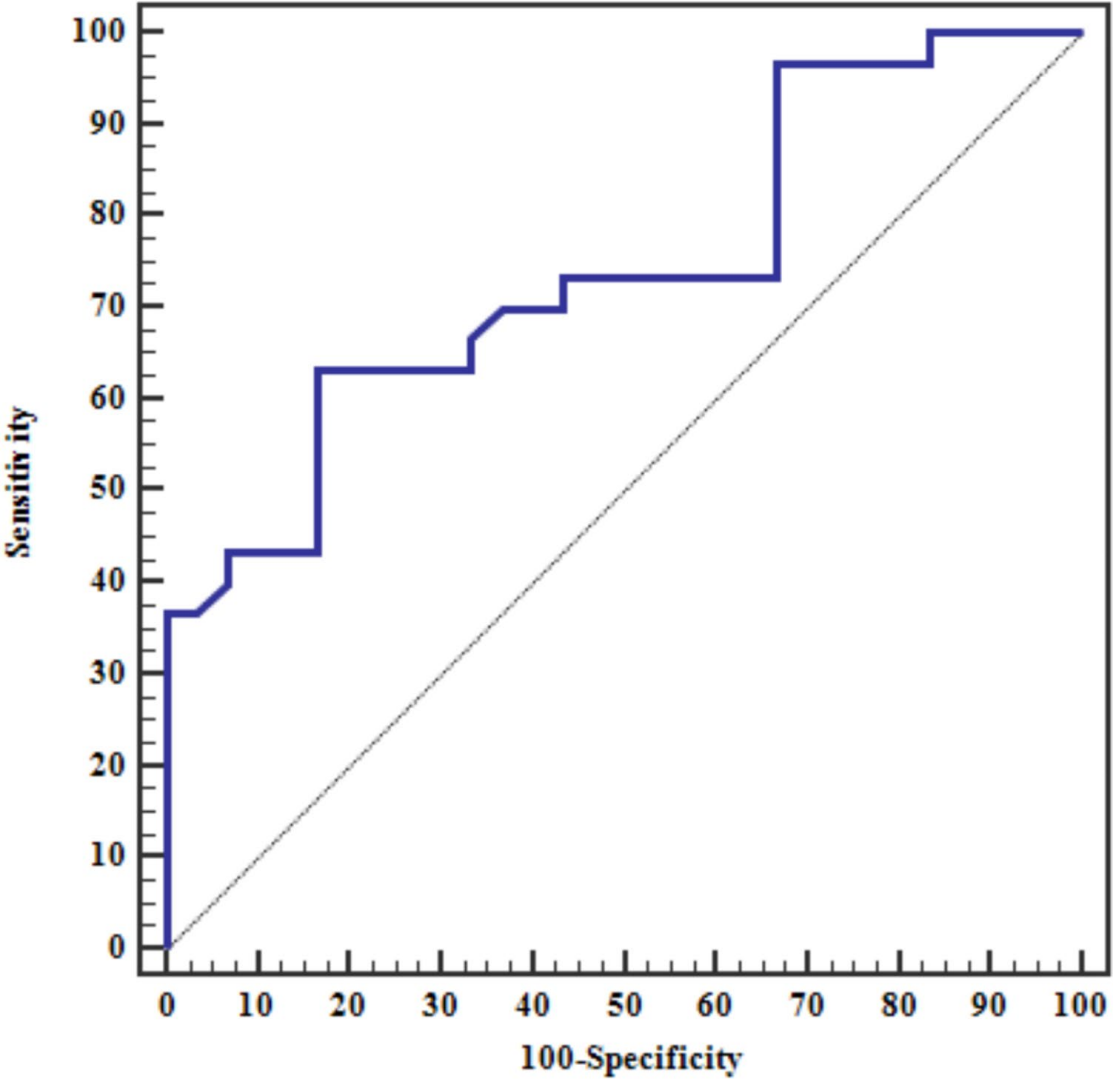
The ROC curve shows that IgA Anti-CD74 has fair discriminatory power, with statistical significance in distinguishing axSpA from peripheral psoriatic arthritis

The diagnostic accuracy for autoantibodies against CD74 in the diagnosis of axSpA revealed that CD74 autoantibodies had 73.33% sensitivity compared to both control groups, 96.67% specificity compared to the healthy control group, and 33.3% specificity compared to the peripheral psoriatic arthritis group (Table 4). The ROC curve shows that IgA Anti-CD74 has excellent discriminatory power, in distinguishing axSpA from healthy controls and fair discriminatory power in distinguishing axSpA from peripheral psoriatic arthritis (Figs. 1, 2).

**Table 4:** Diagnostic performance for IgA Anti-CD74 to discriminate axSpA

Indices	Vs healthy control	vs peripheral psoriatic arthritis control
	Value	
Sensitivity	73.3%	73.3%
Specificity	96.67%	33.3
Positive predictive value	95.7	52.38
Negative predictive value	78.4	55.56
Cutoff point	> 74	> 74
Area under the curve (AUC)	0.984	0.784
P value	< 0.001*	< 0.001*

*AUC* Area Under a Curve

*p* value: Probability value

*Significant *p* value if < 0.05

There was no significant correlation between anti-CD74 IgA antibodies and age, sex, duration of the disease, acute phase reactant, ASDAS-CRP, BASDAI, BASFI, and BASMI (Table 5).

**Table 5:** Correlation between Anti-CD74 and different parameters in axSpA patients (*n* = 30)

	ANTI-CD74	
	r_s_	*p*
Age (years)	−0.069	0.717
Duration of disease (Years)	−0.095	0.617
ASDAS-CPR	0.215	0.254
BASDAI	0.160	0.399
BASFI	0.068	0.721
BASMI	−0.127	0.505
ESR1	0.027	0.888
CRP	0.194	0.304

*r*_s_ Spearman coefficient

Significant *p* value if < 0.05

## Discussion

This study investigates the diagnostic value of anti-CD74 in axSpA and their relation to disease activity.

In this study, the serum levels of anti-CD74 IgA antibodies were significantly higher in axSpA patients compared to both control groups (Peripheral psoriatic arthritis patients and healthy people). 73.3, 66.6 and 3.3% of the axSpA, peripheral psoriatic arthritis, and healthy group, respectively, were anti- CD74 IgA antibody positive based on the cut-off point of anti-CD74 IgA antibodies (> 74 ng/ml). IgA anti-CD74 showed excellent discriminatory power in distinguishing axSpA from healthy controls but interestingly, it showed fair discriminatory power in distinguishing axSpA from peripheral psoriatic arthritis.

This is in agreement with Riechers et al. [13], who reported that IgA anti-CD74 antibodies were higher in the non-radiographic axSpA patients compared to the patients with inflammatory back pain (not fulfilling the criteria of ASAS) controls. IgA anti-CD74 antibodies were found in 47% of non-radiographic axSpA patients, 36% of ankylosing spondylitis, 4.7% of those with inflammatory back pain controls, and 1% in healthy control. They demonstrated that IgA could be a helpful diagnostic tool in early axSpA patients. For detecting non-radiographic axSpA, the specificity was 90% for HLA-B27 and 95.3% for IgA anti-CD74 antibodies. Also, de Winter JJ et al. [19], reported that IgA anti-CD74 antibodies were significantly higher in the axSpA compared to healthy controls and patients with chronic back pain. The frequency of IgA anti-CD74 was 28.5% and 54.7% in r-axSpA and the Spondyloarthritis Caught Early (SPACE) cohort, respectively, compared to 37.0% in patients with chronic back pain. The authors concluded that IgA anti-CD74 antibodies have no diagnostic value in patients with early back pain (LR + was 1.48 and LR- was 0.72).

Do L et al. [20] reported that the plasma level of IgA anti-CD74 antibodies was significantly higher in patients with radiographic axSpA.IgA anti-CD74 antibodies was positive in 23.2%) patients with r-axSpA and 9.9% in controls, with a sensitivity of 0.23 and a specificity of 0.90 and it was associated with radiographic change, supporting its role in the pathogenesis of axSpA.

Previous studies found that anti-CD74 antibodies were significantly higher in patients with SpA than in non-SpA subjects [21–26].

In contrast Çolak S et al. [27] evaluated the level of anti-CD74 in Turkish patients with ankylosing spondylitis and patients with inflammatory bowel disease and found that anti-CD74 levels weren’t higher in patients compared to healthy controls. Also, there is no association between disease activity and the level of anti-CD74.

Kuznetsova D A et al. [28] reported that IgA anti-CD74 was associated with axSpA but not with psoriatic arthritis. However, Baerlecken N T et al. [29] reported that autoantibodies against CD74 were found in 69% of patients with axSpA and 45% of patients with psoriatic arthritis without axial involvement. Therefore, anti-CD74 autoantibodies could be involved in the pathogenesis of SpA, not only in the axial subtype. This finding is in agreement with our result as IgA anti-CD74 was positive in 66.6% of psoriatic arthritis patients without axial affection. Low specificity of IgA anti-CD74 (33.3%) in distinguishing axSpA from peripheral PsA may limit its clinical utility in differentiating axial from peripheral subtype of SpA. More research involving patients with axial SpA and peripheral SpA is required.

Disparities among the various studies could be attributed to multiple factors, including ethnic differences, different cut-off values of IgA anti-CD74 antibodies, the duration of symptoms, and, in some of the studies, the samples were frozen for a long time, which might have affected the results.

The exact cause of IgA anti-CD74 production is still unknown. It is common for the gut to manufacture IgA antibodies. The synthesis of IgA anti-CD74 may therefore be caused by the dysbiosis of the gut flora. The high levels of serum IgA and IgA antibodies against CD74 may be indicators of several subsets of axSpA, each of which has a different set of triggering conditions [13]. Other theories suggested that anti-CD74 antibodies may recognize CD74 degradation fragments that accumulate at the cell surface due to impaired proteolysis [30].

In this study, there was no significant association between IgA anti-CD74 status and HLAB27 status. Also, there was no statistically significant difference between radiographic and non-radiographic axSpA regarding the presence of IgA anti-CD74 and no significant correlation between anti-CD74 IgA antibodies and age, sex, duration of the disease, acute phase reactant, ASDAS-CRP, BASDAI, BASFI, and BASMI. These results are consistent with previous study [22].

It was demonstrated that there was no significant association between IgA anti-CD74 status and HLAB27 status [13, 19, 20, 26]. In contrast previous study by Witte et al. found in one of their two cohorts studied that IgA antibodies against CD74 were found in a higher percentage of HLA-B27 negative patients than HLA-B27positive patients [31]. It was noted that the combination of HLA-B27 and anti-CD74 antibodies showed greater posttest probability and diagnostic value than HLA-B27 alone [13, 23]. This supports the diagnostic value of anti-CD74 antibodies in axial SpA, especially in patients with HLA-B27-negative.

Our results were consistent with De Craemer et al. who found that IgA anti-CD74 did not significantly correlate with age, sex, disease duration, CRP, or disease activity [22]. In contrast to our results, significant correlations were found between IgA anti-CD74 and acute phase reactant [20]. Also it was reported that IgA anti-CD74 showed a significant association with ESR, smoking, and Modified Stoke Ankylosing Spondylitis Score, but no significant associations were found with disease duration, disease activity, BASFI, or BASMI [20]. Additionally, a recent Swiss study reported that elevated IgA anti-CD74 antibodies were associated with older age, male sex, disease duration, BASMI and increased CRP [26].

Also, Witte T et al. reported that there were no significant differences between patients with and without anti-CD74 regarding age, sex, disease duration, BASDAI, and patients with IgA anti-CD74 had higher CRP and Stoke Ankylosing Spondylitis Spine Score (in ENRADAS cohort) than patients without IgA anti-CD74 [31]. Also, de Winter JJ et al. [19] found that sacroiliitis on MRI was associated with elevated IgA anti-CD74. This indicates that IgA anti-CD74 may be associated with structural damage to the spine and sacroiliac joints.

CD74 is considered a T cell antigen in SpA, capable of triggering TH1 and TH17 responses, which are known to be involved in the pathogenesis of axSpA. CD4 + T cells respond to CD74 by producing IL-17A, TNF-α, and IFNγ [32]. CD74 is a receptor for the chemokine macrophage migration inhibitory factor (MIF) [9]. It has been observed that the sera of axSpA patients have greater MIF concentrations than those of healthy controls [10, 31]. The binding of MIF to CD74 triggers signal cascades, including activation of nuclear factor κB, which leads to the production of proinflammatory cytokines. Since MIF activates osteoblasts [11], it could be a contributing factor to the elevated ossification seen in axSpA. higher MIF levels in axSpA patients' sera indicate a quicker course of structural disease [33]. As antibodies against CD74 may activate the same pathways as MIF, they may contribute to the development of axSpA.

Limitations of this study: cross-sectional design limits the ability to determine the causal relation between anti-CD74 antibody and clinical variables & disease activity, and the small sample size. Future studies should focus on long-term research to track the change in anti-CD74 antibodies over time and how they respond to treatment, and to evaluate the level of anti-CD74 in other types of spondyloarthropathies.

## Conclusion

IgA anti-CD74 may be a useful marker for identifying axSpA. Further research on patients with peripheral SpA is required to determine the practical benefits of this biomarker.

## Data Availability

The data will be available upon reasonable request.
